# Endoscopic Management of a Rare Intraluminal Duodenal “Windsock” Diverticulum Causing Recurrent Pancreatitis

**DOI:** 10.5152/tjg.2025.25051

**Published:** 2025-10-31

**Authors:** Oguz Ozturk, Yavuz Cagir, Muhammed Bahaddin Durak, Mucahit Ergul, Ali Atay, Ilhami Yuksel

**Affiliations:** 1 Department of Gastroenterology, Ankara Bilkent City Hospital, Ankara, Türkiye; 2 Department of Gastroenterology, Yenimahalle Training and Research Hospital, Ankara Yıldırım Beyazıt University, Ankara, Türkiye; 3 Department of Gastroenterology, Hacettepe University Faculty of Medicine, Ankara, Türkiye; 4 Department of Gastroenterology, Ankara Yıldırım Beyazıt University Faculty of Medicine, Ankara, Türkiye

## Introduction

Intraluminal duodenal diverticulum, named as windsock diverticulum, is a rare congenital anomaly caused by abnormal duodenal lumen formation due to failed embryonic recanalization. This anomaly may remain asymptomatic or manifest as dyspepsia, gastrointestinal bleeding, or symptoms related to duodenal obstruction, such as nausea, vomiting, and early satiety. In rare instances, especially when the diverticulum extends toward the ampulla of Vater, it may result in biliary or pancreatic duct obstruction, leading to recurrent acute pancreatitis.^1^

## Case Presentation

**Figure 1.**
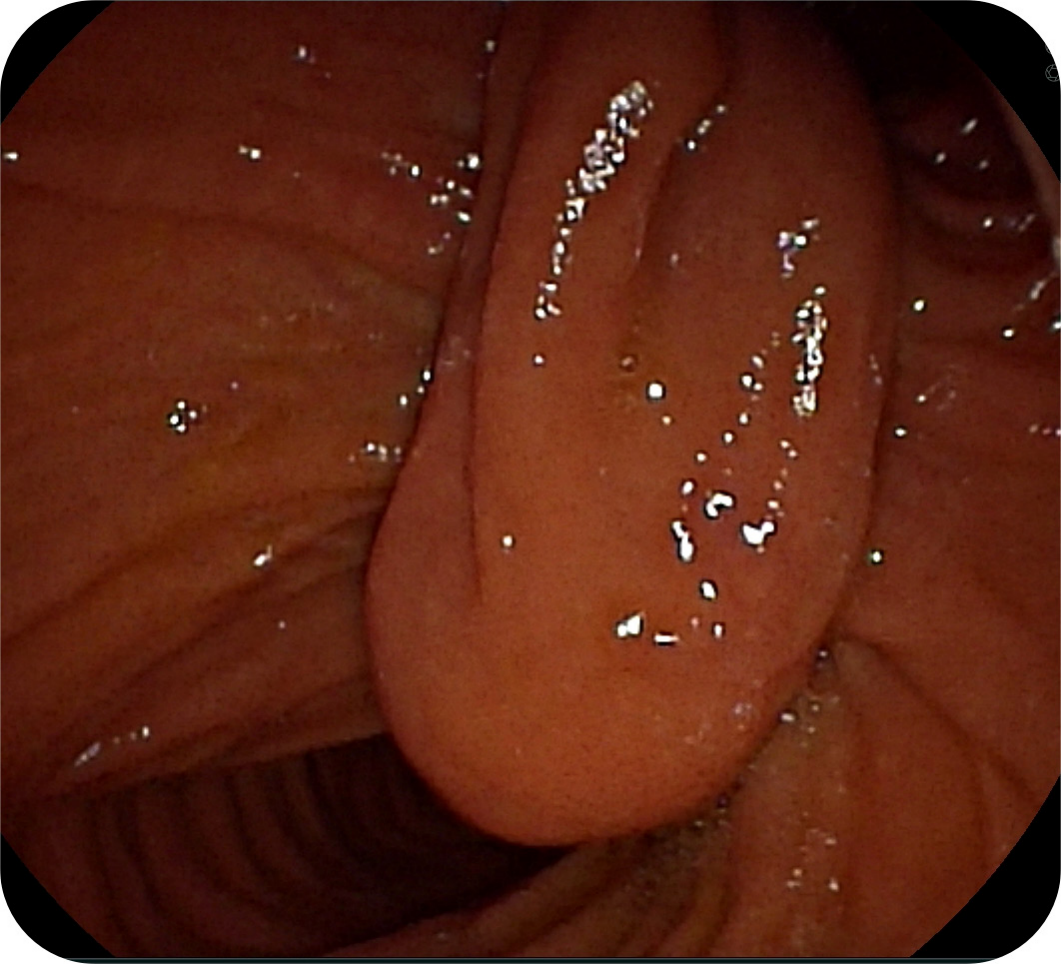
The orifice of the “windsock” diverticulum is characterized by its distinct pouch-like structure, which is clearly separable from the main duodenal lumen. The papilla is not visualized in its typical anatomical location in this view (via side-viewing endoscope).

**Figure 2.**
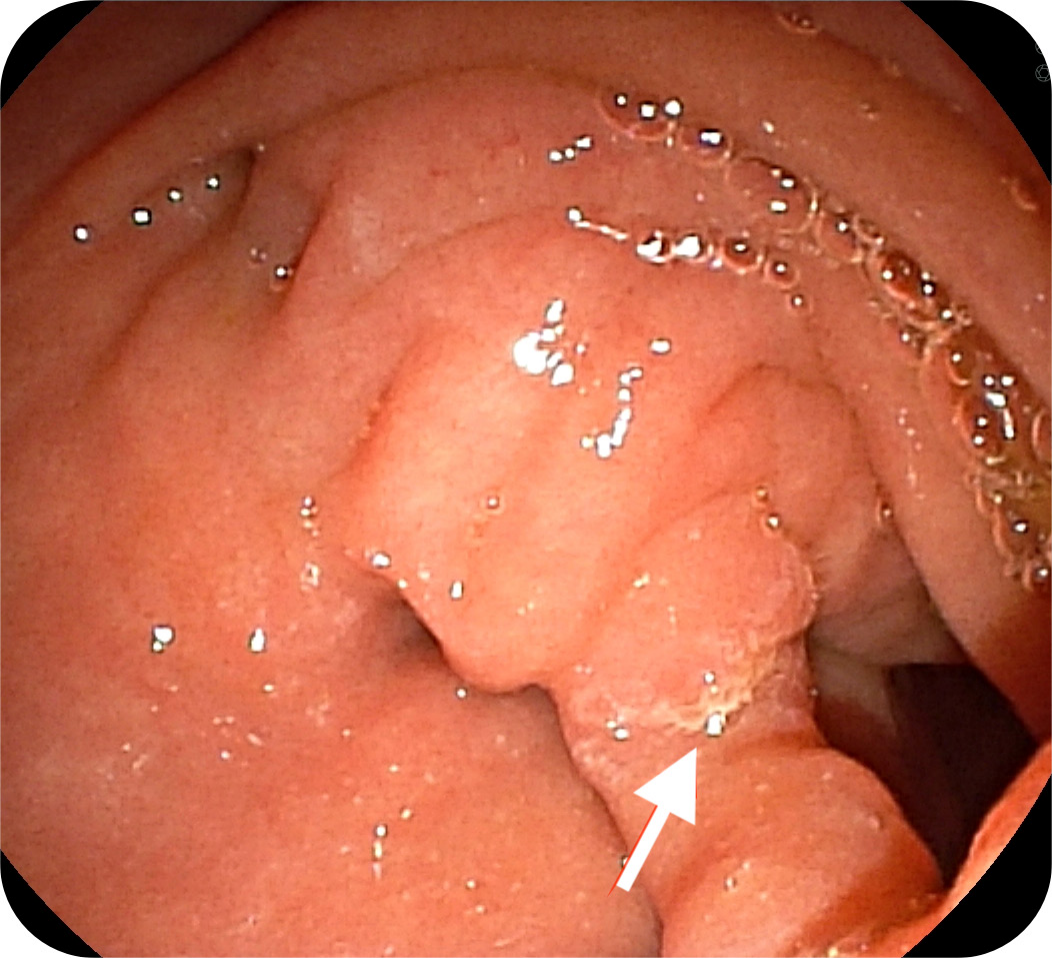
The endoscopic view displays the ampulla of Vater, opening at the septal margin between the diverticulum and the main duodenal lumen (indicated by the white arrow) (via side-viewing endoscope).

A 22-year-old female applied for recurrent acute pancreatitis, having experienced 3 episodes over the previous year. Verbal informed consent was obtained. Her symptoms persisted despite pancreatic duct stenting performed at another institution. Magnetic resonance cholangiopancreatography was utilized to exclude alternative causes such as choledocholithiasis, strictures, or pancreatic masses. Side-viewing endoscopy confirmed a “windsock” diverticulum with the papillary orifice opening at the septal margin between the diverticulum and the duodenum (Figures 1 and 2).

**Figure 3.**
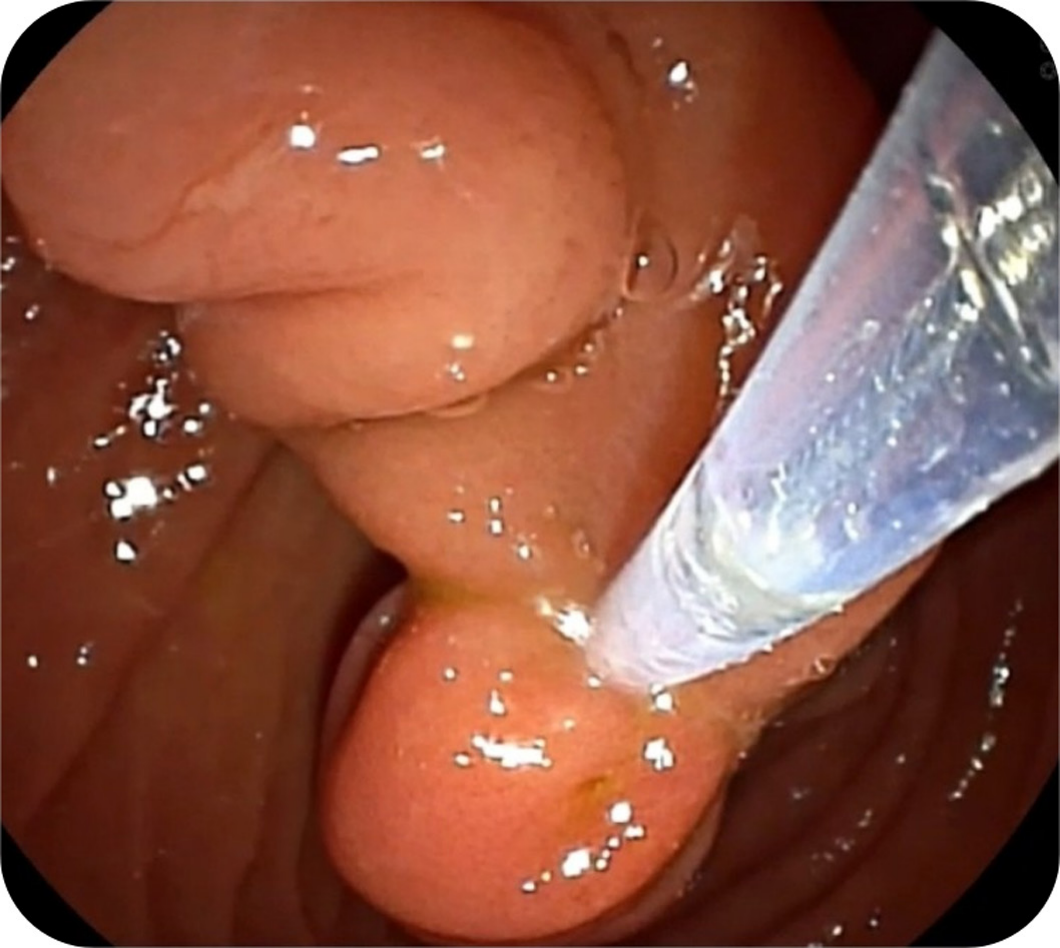
Endoscopic snare resection of the windsock diverticulum (via side-viewing endoscope).

**Figure 4.**
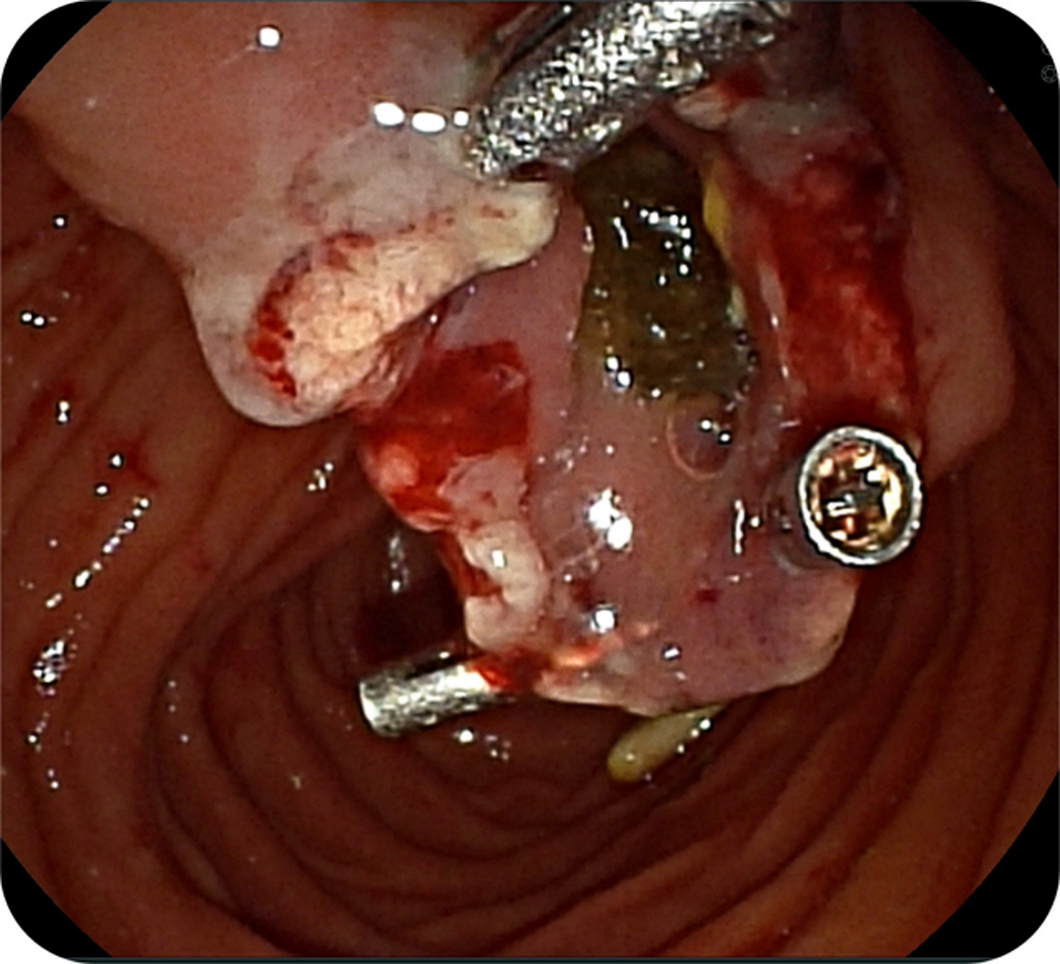
Post-resection area with hemoclips applied to manage hemostasis (via side-viewing endoscope).

Endoscopy revealed an intraluminal bulge separate from the duodenal lumen. A side-viewing endoscope was used to perform snare resection (Figure 3). Oozing bleeding occurred post-resection, and homeostasis was achieved with hemoclips (Figure 4). Histopathological examination confirmed the diagnosis of an epithelial-lined pouch, consistent with a windsock diverticulum, and excluded a duodenal duplication cyst. The patient experienced complete resolution of symptoms with no further episodes for 9 months of follow-up.

## Discussion

The occurrence of pancreatitis as a result of a windsock diverticulum is an uncommon event. The proposed mechanism of the observed phenomenon may be functional ampullary obstruction or diverticulum occlusion with resultant debris accumulation. Another potential causative factor may be dyskinesia, which could result in intermittent blockage and subsequent development of pancreatitis. Biliary obstruction has also been documented in the extant literature.^2^^,^^3^ The differential diagnoses for this condition include the following: periampullary diverticula, duodenal duplication cysts, and congenital duodenal stenosis. It is notable that in the presented case, endoscopic examination from the lateral perspective proved to have a pivotal role in confirming the diagnosis.

The implementation of pancreatic duct stenting can offer symptomatic relief, though repeated procedures may be necessary. The endoscopic procedure with snare resection is a minimally invasive and definitive treatment. Previous reports have indicated favorable results when utilizing this method.^4^ Another recent case of a windsock-shaped intraluminal duodenal diverticulum was reported and treated successfully with endoscopic diverticulectomy, further supporting the safety and efficacy of this technique.^5^

## Conclusion

This case underscores the utility of endoscopic snare resection as a safe and effective treatment for symptomatic windsock diverticulum. Given that endoscopic imaging is essential for diagnosis and therapeutic planning. Endoscopic resection may prevent recurrence, decrease complications, and obviate the need for repeated interventions.

## Data Availability

The data that support the findings of this study are available on request from the corresponding author.
